# Supplementary material for: Development and Clinical Validation of a Skin Test for In Vivo Assessment of SARS-CoV-2 Specific T-Cell Immunity
Source: Viruses. 2025 Aug 29;17(9):1186. doi: 10.3390/v17091186 (PMC12474111; doi:10.3390/v17091186)
Supplement: Supplementary file 1 [file viruses-17-01186-s001.zip › viruses-3824597-supplementary.pdf]

Supplementary file

Data for the integrated analysis were obtained within Phases I–II of the clinical trial conducted according to protocol No. CD-PS-01/21 (single-blind, placebo-controlled study; approved by the Russian Ministry of Health Ethics Council, protocol No. 298 dated 18 January 2022; permission of the Russian Ministry of Health No. 90 dated 10 February 2022) (Groups 1, 2, 3b, 4).

All volunteers who participated in the study met the inclusion criteria and did not present any exclusion criteria, which ensured participant safety and minimized the influence of concomitant somatic conditions.

Inclusion criteria:

- Availability of signed and dated informed consent for participation in the clinical trial prior to any study-related procedures.
- Age ≥18 years.
- Body mass index (BMI) between 18.5 and 34.9 kg/m^2.
- Ability of the volunteer to attend all scheduled visits and complete all study-related procedures as per the study protocol.
- Agreement of the volunteer to either complete sexual abstinence or use an effective method of contraception throughout the study period (until Day 7 after administration of the study product).
- Compliance with one of the following conditions:
  - Vaccinated with EpiVacCorona (according to the Federal Registry of Citizens Vaccinated against COVID-19) ≥14 days and ≤180 days prior to screening.
  - Vaccinated with Gam-COVID-Vac (according to the Federal Registry of Citizens Vaccinated against COVID-19, i.e., having received the second dose of the two-component vaccine during primary vaccination or Sputnik Light in the revaccination regimen) ≥14 days and ≤180 days prior to screening.
  - Vaccinated with CoviVac (according to the Federal Registry of Citizens Vaccinated against COVID-19) ≥14 days and ≤180 days prior to screening.
  - Recovered from COVID-19 infection (i.e., having an epidemiological number in the Federal Service for the Oversight of Consumer Protection and Welfare database) with recovery date ≥14 days and ≤180 days prior to screening, but not vaccinated against COVID-19 (i.e., no record in the Federal Registry of Citizens Vaccinated against COVID-19).
  - No history of COVID-19 infection (i.e., no epidemiological number in the Federal Service for the Oversight of Consumer Protection and Welfare database) and no vaccination against SARS-CoV-2 (for Phase I (Group 1) or Group 4 in Phase II).

Exclusion criteria:

- Age under 18 years.
- Pregnancy or breastfeeding.
- Military personnel or law enforcement officers.
- Persons under custody in pre-trial detention or serving sentences in correctional facilities.
- Contact with confirmed COVID-19 cases within 14 days prior to the screening visit.
- Symptoms of any acute illness at the time of screening.
- Any acute illness resolved less than 4 weeks prior to screening.
- Any acute respiratory tract illness less than 3 months prior to screening.
- Exacerbation of chronic diseases less than 4 weeks prior to study initiation.
- History of hypersensitivity or allergy to any component of the investigational product.

- History of tuberculosis (pulmonary or extrapulmonary), oncological diseases, autoimmune diseases, or dermatological conditions (pemphigus, psoriasis, eczema, atopic dermatitis).
- Long-term use (>14 days) of immunosuppressants, systemic glucocorticosteroids, or immunomodulatory agents within 6 months prior to screening.
- Positive tests for HIV, hepatitis B, hepatitis C, or syphilis.
- Vaccination with SARS-CoV-2 vaccines other than EpiVacCorona, Gam-COVID-Vac, or CoviVac.
  Note: Volunteers vaccinated with Sputnik Light not in the revaccination regimen (i.e., without prior full two-dose vaccination with Gam-COVID-Vac) were not eligible.
- Vaccination with any other vaccine within 1 month prior to screening or planned vaccination during the study.
- Use of immunoglobulin preparations or blood products within 3 months prior to screening.
- Blood or plasma donation (≥450 ml) less than 2 months prior to screening.
- Any other acute or chronic diseases deemed by the investigator to pose safety risks or interfere with compliance and data validity.
- Participation in other clinical trials with an investigational product (or medical device) less than 1 month prior to screening.
- Alcohol, drug, or medication dependence. Consumption of more than 10 units of alcohol per week (1 unit = 500 ml beer, 200 ml wine, or 50 ml vodka) or history of alcoholism, drug abuse, or medication abuse.
- History of psychiatric disorders.
- Failure to meet inclusion criteria.
- For Phase I (Group 1) and for volunteers assigned to Group 4: positive test for SARS-CoV-2 antibodies.

Withdrawal criteria:

Participation of a volunteer in the study could be discontinued if:

- The investigator determined that withdrawal was necessary for medical reasons.
- The volunteer voluntarily withdrew consent for further participation.
- The volunteer was lost to follow-up.
- The volunteer failed to comply with study requirements.
